# Lymphoid and myeloid cell populations in the non-pregnant human Fallopian tube and in ectopic pregnancy

**DOI:** 10.1016/j.jri.2011.01.014

**Published:** 2011-04

**Authors:** J.L.V. Shaw, P. Fitch, J. Cartwright, G. Entrican, J. Schwarze, H.O.D. Critchley, A.W. Horne

**Affiliations:** a Centre for Reproductive Biology, Queen's Medical Research Institute, University of Edinburgh, Edinburgh, UK; b Centre for Inflammation Research, Queen's Medical Research Institute, University of Edinburgh, Edinburgh, UK; c Moredun Research Institute, Pentlands Science Park, Bush Loan, Edinburgh, UK; d The Roslin Institute, Royal (Dick) School of Veterinary Studies, University of Edinburgh, Roslin Biocentre, Midlothian, UK

**Keywords:** Ectopic pregnancy, Fallopian tube, Lymphocytes, Dendritic cells, Monocytes, Neutrophils, Natural killer cells

## Abstract

Lymphoid and myeloid cell populations in human endometrium are well-documented and are known to play important roles in providing immune tolerance, controlling trophoblast invasion, and mediating vascular remodeling. Immune cell populations in the Fallopian tube have not been comprehensively studied. The aim of this study was to characterize lymphoid and myeloid cell populations in non-pregnant Fallopian tube and determine whether they are altered in Fallopian tube from women with ectopic pregnancy. Fallopian tube was analyzed by flow cytometry and immunohistochemistry. Populations of CD3+ (CD4+ and CD8+) lymphocytes, LIN1−HLADR+ (CD123+ and CD11c+) dendritic cells, monocytes, neutrophils, and CD56^dim^CD16− natural killer (NK) cells were demonstrated to be present in non-pregnant Fallopian tube. CD123+ dendritic cells were predominant over CD11c+ dendritic cells. Numbers of CD11c+ cells were significantly higher in the progesterone-dominant mid-luteal phase of the menstrual cycle compared with the follicular phase. Numbers of CD45+ leukocytes, CD68+ cells, and CD11c+ cells were higher in Fallopian tube from women with ectopic pregnancy compared with mid-luteal phase Fallopian tube. These data will advance our understanding of normal human Fallopian tube physiology and disorders of Fallopian tube function, such as ectopic pregnancy.

## 1 Introduction

A number of studies have described lymphoid and myeloid cell populations in the human endometrium and documented hormone-dependent changes during the menstrual cycle and in early pregnancy (Critchley et al., 2001; Laskarin et al., 2007; Salamonsen and Lathbury, 2000; van Mourik et al., 2009). However, lymphoid and myeloid cell populations have yet to be comprehensively studied in the normal human Fallopian tube or in the context of tubal abnormalities, such as ectopic pregnancy.

Populations of leukocytes, including lymphocytes, macrophages, neutrophils, dendritic cells, and uterine (u) NK cells have all been demonstrated in the endometrium (Laskarin et al., 2007; Moffett-King, 2002; Salamonsen and Lathbury, 2000). Greater numbers of CD8+ T lymphocytes than CD4+ T lymphocytes are found in the endometrium and the numbers of endometrial T lymphocytes, particularly CD8+ T lymphocytes, are decreased in the secretory phase compared with the proliferative phase of the menstrual cycle (Flynn et al., 2000). Macrophages, dendritic cells and uNK cells infiltrate the endometrium under the influence of progesterone during the late-secretory phase, following ovulation and during early pregnancy (Critchley et al., 2001; Dunk et al., 2008; King, 2000). CD3−CD56^bright^CD16− uNK cells dominate the decidualized endometrium during early pregnancy (Le and Piccinni, 2008; Moffett-King, 2002). These cells differ from peripheral blood NK cells, which are usually CD3−CD56^bright^CD16+ and have cytotoxic properties (Le and Piccinni, 2008). Endometrial NK cells are less cytotoxic than peripheral blood NK cells; they are cytokine-producing and have receptors capable of interacting with the trophoblast (Moffett-King, 2002). This interaction helps to control vascular remodeling and regulate trophoblast invasion (Le and Piccinni, 2008; Makrigiannakis et al., 2008; Moffett-King, 2002; von Rango, 2008). Macrophages, dendritic cells, and lymphocytes are also present in the uterus during pregnancy (Nagamatsu and Schust, 2010). Macrophages play roles in tissue remodeling and angiogenesis and are important for cleaning up apoptotic trophoblast cells to prevent activation of T cells (Nagamatsu and Schust, 2010; van Mourik et al., 2009). Decidual dendritic cells are also believed to direct a tolerogenic response toward the semiallogenic embryo by regulating the phenotype of T cells within the endometrium (Blois et al., 2007; Laskarin et al., 2007).

Studies examining immune cell populations and their functions in the Fallopian tube are limited. However, results from immunohistochemical analyses have reported that lymphocytes and macrophages are the most abundant immune cells present in the Fallopian tube (Vassiliadou and Bulmer, 1998). A study using flow cytometry also identified a population of neutrophils in human Fallopian tube (Smith et al., 2006). In contrast to the endometrium, numbers of CD8+ lymphocytes have been shown to be elevated during the mid-luteal phase of the menstrual cycle in the Fallopian tube (Ulziibat et al., 2006), suggesting that tubal immune cells may also be influenced by hormonal changes during the menstrual cycle, but may be regulated differently than endometrial immune cells. In Fallopian tube from women with ectopic pregnancy, CD3−CD56^bright^CD16− NK cells have been shown to be absent from the tubal implantation site (Proll et al., 2000; Vassiliadou and Bulmer, 1998; von Rango et al., 2001). However, a population of CD3−CD56^dim^CD16− NK cells has recently been reported in Fallopian tube from women with ectopic pregnancy (Laskarin et al., 2010). The numbers of CD8+ lymphocytes and macrophages have been shown to be elevated at the tubal implantation site compared with elsewhere in the same Fallopian tube (Ulziibat et al., 2006; von Rango et al., 2001).

The aim of this study was to characterize the lymphoid and myeloid cell populations present in non-pregnant Fallopian tube collected from women throughout the menstrual cycle and in Fallopian tube from women with ectopic pregnancy. There are few data available describing the dendritic cell (DC) or NK cell phenotypes within the Fallopian tube, although previous studies have demonstrated several leukocyte populations in the Fallopian tube. This study will focus, in particular, on the characterization of DC and NK cell phenotypes.

## 2 Materials and methods

### 2.1 Collection of tissue samples

Ethical approval for this study was obtained from the Lothian Research Ethics Committee (LREC 04/S1103/20) and informed consent was obtained from all the women participating in the study. Fallopian tube biopsies (2–3 cm in length) from the ampullary region were collected at the time of hysterectomy (*n* = 13) and at the time of surgical management for ectopic pregnancy (*n* = 5). Women were between 18 and 45 years of age and the clinical details for each participant are listed in Tables 1 and 2. The biopsies were divided into two portions: either stored in phosphate-buffered saline (PBS), for a maximum of 2 h, prior to preparation for flow cytometric analysis (see below), or fixed in 4% neutral-buffered formalin overnight at 4 °C followed by storage in 70% ethanol, and subsequent embedding in paraffin wax for immunohistochemical staining. Menstrual cycle dating was determined by three criteria, each of which had to correlate in order for the sample to be included in the study: (1) date of their last menstrual period (as reported by the patient); (2) staging by an expert gynecological pathologist of an endometrial biopsy obtained at the time of Fallopian tube biopsy; and (3) estradiol and progesterone measurements.

### 2.2 Flow cytometry

The Fallopian tube was finely dissected, minced and digested using 1 mg/mL collagenase IV (Sigma–Aldrich, Dorset, UK) and 1 mg/mL DNase (Sigma–Aldrich) in PBS for 2 h at 37 °C. Digested tissue was passed through a 19 G needle repeatedly, following which cells were filtered through 70 μm and 40 μm filters. Filtered material contained a single cell suspension, which was centrifuged for 3 min at 1700 rpm. Cells were resuspended in 1.5 mL of flow cytometry wash buffer (1% BSA in PBS) containing 1% normal mouse serum prior to staining. Cells (50 μL of suspension) were stained with the following mouse monoclonal antibodies and isotype controls, all from BD Biosciences (Oxford, UK): anti-CD3 APC (clone M-A712), anti-CD4 (sk3) PE, anti-CD8 (sk1) FITC, anti-CD19 (SJ25C1) PERCP, anti-LIN1 FITC, anti-HLADR (g 46-6) PERCP, anti-CD11c (shcl-3) APC, anti-CD123 (9f5) PE, anti-CD14 PERCP (mφp9), anti-CD16 (3g8) FITC, anti-CD56 (NCAM 16.2) PE, mouse IgG1 FITC (X40), mouse IgG2b FITC (27-35), mouse IgG2a PERCP (X39), mouse IgG2b PE (27-35). Cells were stained for 30 min and fixed after red blood cell lysis with FACs^®^ lysing solution (BD Biosciences, Oxford, UK). Samples were analyzed using a BD FACsCalibur^®^ flow cytometer and FACs DIVA software (BD Biosciences).

### 2.3 Immunohistochemistry

Immunohistochemistry was performed on 3-μm thick paraffin-embedded sections using standard techniques (Battersby et al., 2004). Sections were blocked in normal goat serum diluted 5-fold in 5% bovine serum albumin and Tris-buffered saline was used as an antibody diluent. Anti-human CD45 mouse monoclonal antibody (Dako, Cambridge, UK) was diluted 50-fold, anti-human CD68 mouse monoclonal antibody (Dako, Cambridge, UK) was diluted 500-fold, and anti-human CD11c rabbit polyclonal antibody (Abcam, Cambridge, UK) was diluted 100-fold. In all cases negative controls were performed using polyclonal mouse IgG1 at the same concentration as the primary antibody, except for CD11c, in which case the absence of primary antibody was used as the negative control.

### 2.4 Stereology

Following immunohistochemistry stained cells were quantified. For each section analyzed, the software used (Image-Pro Plus stereology software version 6.2, Media Cypernetics Inc., Bethesda, MD, USA) prepared 200 grids (14 μm × 14 μm) spanning the entire section of tissue. DAB-stained cells were counted in grids chosen by the software at random using 40× magnification. Cells were counted until a significant number of cells had been counted, as per the software specifications. The number of stained cells per grid counted was then calculated.

### 2.5 Statistical analysis

Analysis was performed using GraphPad Prism (version 5.0, GraphPad Software, La Jolla, CA, USA). Where three groups were compared, differences between groups were assessed using one-way ANOVA followed by post hoc analysis by the Newman–Keuls test. For assessment of differences between two groups, unpaired *T*-tests were performed. Results were considered significant if *p* < 0.05.

## 3 Results

### 3.1 Analysis of immune cell populations in non-pregnant human Fallopian tube by flow cytometry

**Fig. 1:**
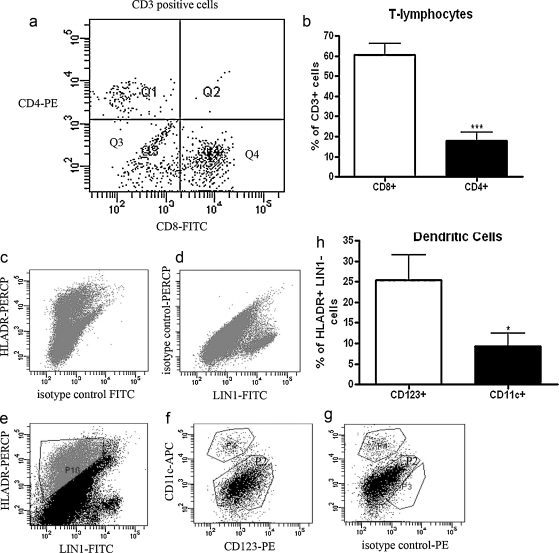
Analysis of lymphocyte and dendritic cell populations in human Fallopian tube by flow cytometry. Fallopian tube biopsies (*n* = 5) were minced and digested to form a single cell suspension. Cells were stained with antibodies in combination to analyze specific populations of cells. (a) Cells positive for staining with APC-conjugated CD3 were gated and are shown as either CD4+ (Q1) or CD8+ (Q4) T cells. (b) Percentages of CD3+CD4+ and CD3+CD8+ T cells. (c) Cells positively staining for HLADR-PERCP. (d) Cells positively staining with a combination lineage marker 1 (LIN1)-FITC. (e) Cells positively stained for HLADR and not stained with LIN1 are shown in P10. (f) HLADR+LIN1− cells were gated and populations of HLADR+LIN1−CD123+ and HLADR+LIN1−CD11c+ subpopulations are shown in P3 and P4 respectively. (g) Isotype control for CD123 antibody. (h) Percentages of LIN1−HLADR+ cells that were CD123+ or CD11c+. P2 represents cells that were HLADR+LIN1−, but negative for CD11c or CD123. These cells likely include epithelial cells.

**Fig. 2:**
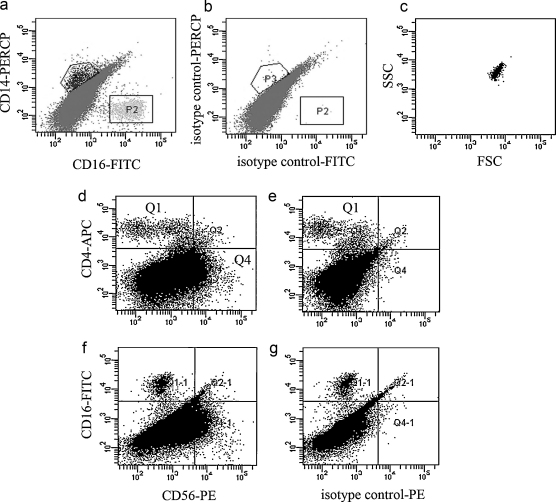
Analysis of monocyte and NK cell populations in human Fallopian tube by flow cytometry. Fallopian tube biopsies (*n* = 5) were minced and digested to form a single cell suspension. Cells were stained with antibodies in combination to analyze specific populations of cells. (a) Populations of live cells staining with CD16 (P2) or CD14 (P3). (b) Isotype control for CD16 and CD14 antibodies. (c) Cells positively stained for CD16 were gated and are shown in the plot as forward scatter versus side-scatter to demonstrate the degree of granularity of these cells. (d) The population of CD3−CD56^dim^ cells are shown in Q4. (e) Isotype control for CD56 antibody. (f) Plot shows that the population of CD56^dim^ cells are CD16−. (g) Isotype control for CD56 shown with CD16.

Lymphocyte, dendritic, monocyte and NK cell populations were analyzed in Fallopian tube from non-pregnant women (*n* = 5). T lymphocytes were defined as CD3+ and either CD4+ or CD8+. There were populations of both CD3+CD4+ cells and CD3+CD8+ cells (Fig. 1a) with a significantly higher percentage of CD8+ than CD4+ cells (Fig. 1b). Dendritic cells (DC), which express HLA-DR, but not the combination of lineage markers (LIN1) used, were found in Fallopian tube (Fig. 1c–e) and both subpopulations of LIN1−HLADR+CD123+ plasmacytoid DCs (pDC) and LIN1−HLADR+CD11c+ conventional DCs (cDC) were present (Fig. 1f and g). A significantly higher percentage of the LIN1−HLADR+ cells expressed CD123+ than CD11c (Fig. 1h), indicating that pDCs are more prevalent than cDCs in Fallopian tube. CD16 and CD14 were used to analyze the populations of monocytes and granulocytes (Fig. 2a and b). Both CD16+CD14− cells and CD14+CD16− cells but no double positive populations were detected. Analysis of CD16+ cells gated by forward and side scatter (Fig. 2c) showed that these cells were granular in nature and therefore were likely to be neutrophils. The CD14+CD16− cells were regarded as monocytes. Only a very small population of CD3−CD56^bright^CD16− NK cells was found in the non-pregnant Fallopian tube, while a population of CD3−CD56^dim^ NK cells represented approximately 10% of all live cells (Fig. 2d and e). More than 90% of CD3−CD56^dim^ cells did not express CD16 (Fig. 2f and g).

### 3.2 Immune cell numbers in Fallopian tube at different stages of the menstrual cycle and in ectopic pregnancy

**Fig. 3:**
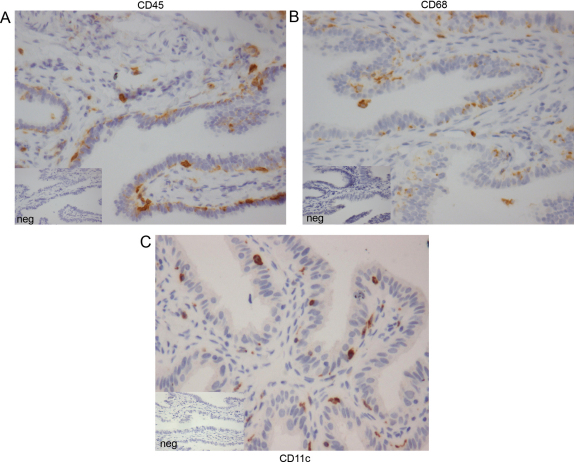
Immunohistochemical localization of CD45+, CD68+, and CD11c+ cells in human Fallopian tube. (a) CD45+ leukocytes, (b) CD68+, and (c) CD11c+ cells localized to the epithelium of human Fallopian tube taken from the mid-luteal phase of the menstrual cycle.

**Fig. 4:**
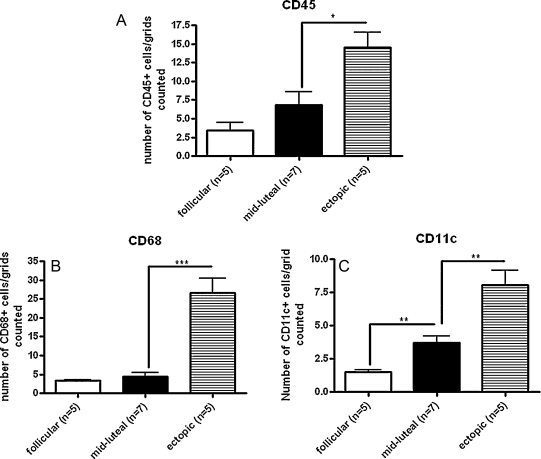
Quantification of CD45+, CD68+, and CD11c+ cells in Fallopian tube from women across the menstrual cycle and from women with ectopic pregnancy. Cells stained positively for CD45 (a), CD68 (b) or CD11c (c) by immunohistochemistry were quantified using stereology software.

Tissue sections of Fallopian tube (*n* = 13) were stained by immunohistochemistry for the immune cell markers. CD45+ (Fig. 3a), CD68+ (Fig. 3b) and CD11c+ (Fig. 3c). Cells expressing these leukocyte markers were distributed at the epithelial surface immediately subjacent to the epithelial cell layer in Fallopian tube from non-pregnant women (Fig. 3) and from women with ectopic pregnancy (data not shown). Immune cells in these sections were quantified using stereology software. There were significantly higher numbers of CD11c+ myeloid cells (*p* < 0.01), but no difference in CD45+ leukocytes or CD68+ macrophages, in Fallopian tube from the progesterone-dominant mid-luteal phase of the menstrual cycle compared with that from the follicular phase (Fig. 4a–c). In Fallopian tube from women with ectopic pregnancy, the numbers of all CD45+ cells (*p* < 0.05), CD68+ cells (*p* < 0.0001), and CD11c+ cells (*p* < 0.01) were significantly increased compared with the mid-luteal phase of the menstrual cycle (Fig. 4a–c). Owing to ethical constraints, it is not possible to collect Fallopian tube from women with intrauterine pregnancies. Therefore, Fallopian tube collected from the mid-luteal phase, when circulating progesterone levels are increased, provides the most appropriate control.

## 4 Discussion

We present a comprehensive survey of the immune cell populations present in human Fallopian tube, using flow cytometry and immunohistochemistry. We demonstrate for the first time that the human Fallopian tube is populated by CD123+ plasmacytoid DCs and CD11c+ conventional DCs and that the non-pregnant Fallopian tube contains a population of CD3−CD56^dim^CD16− NK cells. We report that plasmacytoid DCs are the dominant dendritic cell population in the Fallopian tube. Furthermore, we show that numbers of CD11c+ cells, which include dendritic cells, are increased in the Fallopian tube during the mid-luteal phase of the menstrual cycle compared with the follicular phase. In addition, we report that numbers of CD45+ leukocytes, CD68+ cells, likely to be macrophages and CD11c+ cells are significantly increased in Fallopian tube from women with ectopic pregnancy compared with mid-luteal phase Fallopian tube. We confirm previous findings indicating the presence of T lymphocytes, predominantly CD8+ T cells (Ulziibat et al., 2006; Vassiliadou and Bulmer, 1998), neutrophils, and macrophages (Gaytan et al., 2007; von Rango et al., 2001) in the non-pregnant human Fallopian tube.

There are two main lineages of human dendritic cells, conventional and plasmacytoid. Conventional dendritic cells express CD11c+, whereas CD123+ is expressed by plasmacytoid dendritic cells (Robinson et al., 1999). CD11c+ conventional dendritic cells produce cytokines important for the development of T-helper cell responses (Lebre and Tak, 2009). CD123+ plasmacytoid dendritic cells can be activated by viruses or bacteria and produce large amounts of interferon alpha (IFNα) (Mellor and Munn, 2004). CD123+ plasmacytoid dendritic cells have also been shown to induce tolerance through IFNα production, by suppressing T cell proliferation and inducing regulatory T cells (Park et al., 2008). Using flow cytometry, we found a relatively large percentage of CD123+ plasmacytoid dendritic cells in human Fallopian tube and a significantly higher percentage of CD123+ dendritic cells compared with CD11c+ dendritic cells. Given that the Fallopian tube is exposed to allogeneic spermatozoa and the semiallogeneic embryo and must exhibit tolerance toward these species for successful reproduction, plasmacytoid dendritic cells may play a role in promoting a tolerant phenotype within the tubal environment.

In Fallopian tube sections, we found a higher percentage of CD11c+ cells during the progesterone-dominant mid-luteal phase of the menstrual cycle compared with the follicular phase. These findings suggest that tubal immune cells may be regulated by hormonal changes similar to what is observed in the endometrium. During pregnancy, dendritic cells have the ability to interact with fetal trophoblast cells and are thought to limit trophoblast invasion in the endometrium (Laskarin et al., 2007; Moffett-King, 2002; Renaud et al., 2007). It is possible that the higher numbers of CD11c+ cells found in the Fallopian tube during the mid-luteal phase play a role in preventing trophoblast invasion of the Fallopian tube. It would be interesting to characterize CD11c+ cells from Fallopian tube of women with ectopic pregnancy and compare them with CD11c+ cells from pseudopregnant or non-pregnant women. During the menstrual cycle, it is likely that CD11c+ cells are present during the mid-luteal phase so that they may respond to foreign pathogens that may have entered with the seminal fluid, protecting the Fallopian tube and embryo from infection.

When implantation does occur in the Fallopian tube, it is characterized by uncontrolled invasion of the tubal wall by trophoblast cells, leading to life-threatening tubal rupture (Varma and Gupta, 2009). In the endometrium, trophoblast invasion and implantation are tightly controlled and this control is attributed to the large numbers of cytokine-producing CD3−CD56^bright^CD16− uNK cells present (Le and Piccinni, 2008; Moffett-King, 2002). uNK cells interact with trophoblast through specific receptors and these interactions are believed to result in the production of cytokines by uNK cells, which affect trophoblast behaviour (Norwitz et al., 2001). CD56^bright^ NK cells have previously been reported to be absent from the non-pregnant Fallopian tube (Vassiliadou and Bulmer, 1998) a finding confirmed by our data. These cells are also absent in Fallopian tube from ectopic pregnancy (Vassiliadou and Bulmer, 1998; von Rango et al., 2001). However, similar to a recent study (Laskarin et al., 2010), we found substantial numbers of CD3−CD56^dim^CD16− NK cells in Fallopian tube from non-pregnant women. Little is known about the function of this NK cell subtype and in the peripheral blood these cells account for less than 1% of all mononuclear cells (Lanier et al., 1986). However, one study found that when peripheral blood derived CD56^dim^CD16− NK cells were treated with pro-inflammatory cytokines, they proliferated and developed into CD56^bright^CD16+ cells with cytotoxic properties (Takahashi et al., 2007). The same study also demonstrated that CD56^dim^CD16− cells proliferate and produce interferon-gamma when exposed to *Streptococcus pyogenes*-stimulated monocytes. These data suggest that CD56^dim^CD16− cells may play roles in immunity against pathogens. The role of this NK cell subtype in human Fallopian tube can only be postulated and further studies are required.

We demonstrate significantly higher numbers of CD11c+ cells, likely dendritic cells, in Fallopian tube from women with ectopic pregnancy compared with mid-luteal phase Fallopian tube. Unfortunately, staining with a CD123 antibody was unsuccessful and we were unable to quantify CD123+ cells in the Fallopian tube by immunohistochemistry. Similar to a previous report (von Rango et al., 2001), we show that numbers of CD68+ cells, likely to be macrophages, are significantly increased in Fallopian tube from women with tubal ectopic pregnancy. Decidual dendritic cells are known to express indoleamine 2,3 dioxygenase (IDO), the rate-limiting enzyme involved in tryptophan catabolism (Miwa et al., 2005). IDO-expressing trophoblast, macrophages and dendritic cells have been shown to inhibit T cell proliferation thereby inducing tolerance (Munn et al., 1998, 1999). It is possible that tubal dendritic cells are also important for the induction of tolerance. However, the higher numbers of immune cells present in the Fallopian tube during ectopic pregnancy could simply be a reflection of the increased hormone levels present during pregnancy. It is also likely that invasion of the tubal wall by trophoblast results in inflammatory signals causing an influx of immune cells to the Fallopian tube.

We acknowledge several limitations to the current study. Fallopian tube biopsies are difficult to obtain and as such, a limited number of tissues were available for analysis by flow cytometry. Studies using large numbers of tissues are necessary to confirm the findings presented here. In addition, these results may not be reflective of immune cell populations present within the entire Fallopian tube, as only the ampullary section was studied. Unfortunately, we were unable to study the entire Fallopian tube from each patient as it was an ethical requirement that the majority of the tube had to be sent for routine histopathology.

In summary, we show that the human Fallopian tube is populated by immune cells of myeloid and lymphoid lineages. We present novel findings demonstrating the presence of dendritic cells and CD56^dim^CD16− NK cells in the non-pregnant Fallopian tube and report that the population of immune cells is altered in the mid-luteal phase of the menstrual cycle, which may indicate progesterone regulation of these cells. We also demonstrate differences in the numbers of myeloid cells in Fallopian tube from women with ectopic pregnancy compared with the non-pregnant Fallopian tube. These data are important for furthering our understanding of normal human Fallopian tube immunology and the etiology of disorders affecting Fallopian tube function, such as ectopic pregnancy.

## Figures and Tables

**Table 1:** Clinical information for Fallopian tube samples across the menstrual cycle.

Sample	Cycle phase	Serum estrogen (pmol/L)	Serum progesterone (nmol/L)	Reason for surgery
1	Follicular	1023	0.8	HMB
2	Follicular	940	3.8	HMB, PP
3	Follicular	829	4.2	HMB, PP
4	Follicular	771	5.2	HMB
5	Follicular	116	2.9	HMB, PP
6	Follicular	55	2.2	HMB, PP
7	Mid-luteal	550	88.0	Dysmen
8	Mid-luteal	242	53.1	HMB, dysmen
9	Mid-luteal	201	24.6	HMB, PP
10	Mid-luteal	400	80.5	HMB, dysmen
11	Mid-luteal	1633	54.4	HMB, dysmen
12	Mid-luteal	424	76.9	PP
13	Mid-luteal	243	38.1	HMB

HMB = heavy menstrual bleeding; PP = pelvic pain; dysmen = dysmenorrhea.

**Table 2:** Clinical information for Fallopian tubes from ectopic pregnancy.

Sample	hCG (IU/L)	Serum progesterone (nmol/L)	Gestational age (days)
1	5981	158.1	41
2	453	8.8	47
3	10,285	31.7	46
4	1082	23.9	52
5	508	7.1	44
